# The Role of the Signaling Pathways Involved in the Effects of Hydrogen Sulfide on Endoplasmic Reticulum Stress

**DOI:** 10.3389/fcell.2021.646723

**Published:** 2021-03-19

**Authors:** Shizhen Zhao, Xinping Li, Ping Lu, Xiaotian Li, Mingfei Sun, Honggang Wang

**Affiliations:** ^1^Institute of Biomedical Informatics, Bioinformatics Center, School of Basic Medical Sciences, Henan University, Kaifeng, China; ^2^Key Laboratory of Natural Medicine and Immuno-Engineering, Henan University, Kaifeng, China; ^3^The First Affiliated Hospital of Henan University, Kaifeng, China

**Keywords:** hydrogen sulfide, endoplasmic reticulum stress, signaling pathway, unfolded protein response, disease treatment

## Abstract

Endoplasmic reticulum (ER) is a kind of organelle with multiple functions including protein synthesis, modification and folding, calcium storage, and lipid synthesis. Under stress conditions, ER homeostasis is disrupted, which is defined as ER stress (ERS). The accumulation of unfolded proteins in the ER triggers a stable signaling network named unfolded protein response (UPR). Hydrogen sulfide is an important signal molecule regulating various physiological and pathological processes. Recent studies have shown that H_2_S plays an important role in many diseases by affecting ERS, but its mechanism, especially the signaling pathways, is not fully understood. Therefore, in this review, we summarize the recent studies about the signaling pathways involved in the effects of H_2_S on ERS in diseases to provide theoretical reference for the related in-depth researches.

## Introduction

Endoplasmic reticulum (ER) is an important organelle controlling proteins synthesis, modification and folding, calcium storage and lipid synthesis (Jeong et al., 2017; So, 2018; Park et al., 2019). It also contributes significantly to cell structure/function by producing the most of the membrane lipids for other organelles (Guzel et al., 2017). Under stress conditions such as viral infection, environmental toxins, glucose deficiency, oxidative stress, hypoxia, inflammation, and changes in Ca^2+^ levels, ER homeostasis is disrupted, which is defined as ER stress (ERS). ERS is a disorder of ER function, which interferes with proteins folding, secretion and post-translational modification. At last, the accumulation of unfolded proteins in the ER triggers a stable signaling network named unfolded protein response (UPR) (Wang et al., 2020). In some cases, ERS and UPR protect cells against stress and contribute to restoring intracellular homeostasis. However, the prolonged activation of the UPR is associated with many pathologies, including metabolic diseases, viral infections, neurodegenerative diseases, cancer, and ophthalmic diseases (Fernandez et al., 2015; Cakir and Nillni, 2019; Ji et al., 2019). Up to now, the exact mechanism of ERS in diseases is not fully understood.

Hydrogen sulfide (H_2_S) is an important signaling molecule that regulates many physiological and pathological processes (Yang et al., 2020). Recent studies have shown that the effects of H_2_S on ERS play an important role in many diseases including neurological diseases, respiratory diseases, vascular disease (Wang et al., 2020). The above processes involve a variety of signaling pathways, and the relevant researches are helpful to explore the mechanism of the effects of H_2_S on ERS in diseases. Therefore, in this review, we summarize the recent studies about the signaling pathways involved in the effects of H_2_S on ERS in diseases to provide theoretical reference for the related in-depth researches.

## Overview of Ers

Endoplasmic reticulum (ER) is a reticular organelle composed of tubular structure and flat sac and it is composed of smooth and rough ER. Smooth ER is responsible for the production and synthesis of structural and non-structural fatty acids and phospholipids. In addition, it plays an important role in calcium homeostasis and carbohydrate metabolism. Rough ER is the main site for proteins synthesis, modification, proteins folding and assembly into stable secondary and tertiary structures, as well as the secretion of proteins (Young, 2017). The various stressors including hypoxia, hypoglycemia, stress, calcium deficiency, high-fat diet, and oxidative stress, can disturb the protein folding process, resulting in the accumulation of unfolded and misfolded proteins in the ER, which is called ERS (Mao et al., 2019). The accumulation of unfolded proteins in ER triggers a stable signal network called UPR to reduce the overload caused by unfolded protein. One of the mechanisms of the above effects is that UPR can activate a series of signals to promote the synthesis of new proteins in response to stress, and reduce the synthesis of general proteins. Another is that UPR can promote protein degradation through autophagy and enhance the clearance of unfolded proteins through a process called ER-associated degradation (Iurlaro and Munoz-Pinedo, 2016). If ER function is seriously damaged, ERS can induce apoptosis (Hetz et al., 2015; Chan et al., 2016). The apoptosis induced by ERS is mainly achieved by the following three pathways: the activation of transcription factor C/EBP homologous protein (CHOP)/growth inhibition and DNA damage induced gene 153 (*Gadd153*), the activation of apoptosis signal-regulating kinase 1(ASK1)/c-Jun N-terminal kinase (JNK) kinase pathway and the activation of caspase 12 (Wu et al., 2018b).

**FIGURE 1:**
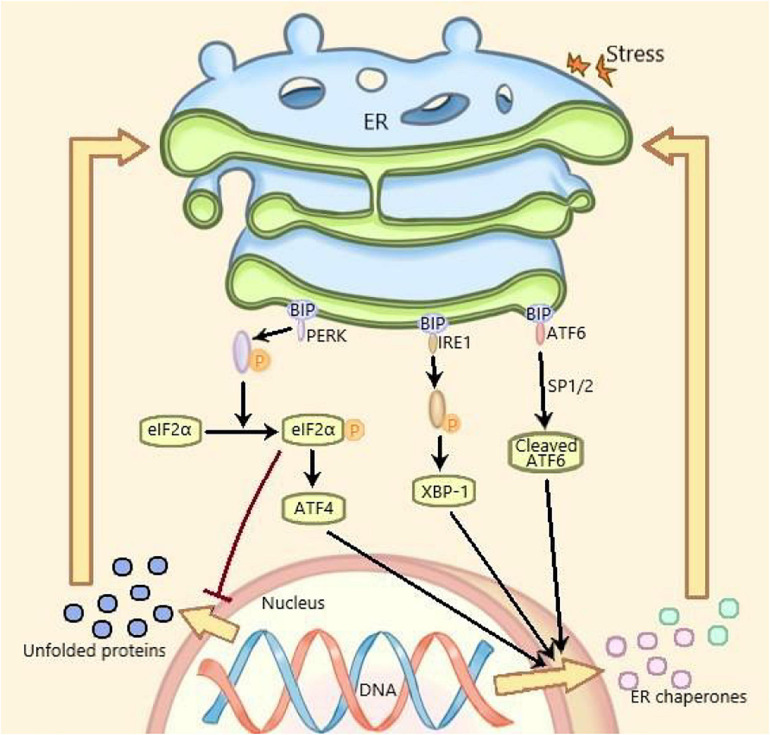
ERS and UPR are mediated by three parallel signal transduction pathways. Binding immunoglobulin (BIP) binds to PERK, IRE1, and ATF6 to stabilize and prevent their activation under non-pressure conditions. The stressors and the unfolded proteins promote the separation of BiP from PERK, IRE1, and ATF6, thus activating these three molecules. Subsequently, the self phosphorylated PERK phosphorylated eIF2a, inhibited mRNA translation and protein synthesis, increased the expression of ATF4. The activated IRE1 cleaved XBP1 mRNA. The isolated ATF6 was cleaved by the 1-site protease (sp1) and 2-site protease (sp2) proteins of Golgi complex. Finally, the cleaved XBP1, ATF4, and spliced ATF6 promote the expression of Er chaperone genes, which are further involved in the elimination of unfolded proteins and the restoration of normal cell homeostasis. PERK, pancreatic endoplasmic reticulum kinase; IRE1, inositol-requiring enzyme 1; ATF6, activating transcription factor 6; XBP1, X-box binding protein 1; ERS, endoplasmic reticulum stress; UPR, unfolded protein response.

Under ERS, the protein expression level of GRP78, ATF6, phosphor-PERK, and phosphor-IRE1 are increased and considered as the markers of ERS (Su and Li, 2016). ERS is mediated by three ERS sensors: pancreatic endoplasmic reticulum kinase (PERK), inositol-requiring enzyme 1 (IRE1) and activating transcription factor 6 (ATF6), which, respectively, mediates one of three parallel signal transduction pathways (Lindahl et al., 2017). Under non-stressful conditions, the binding immunoglobulin (BIP) binds to PERK, IRE1, and ATF6 to stabilize and prevent their activation. The stressors leading to ERS and the unfolded proteins promote the isolation of BIP from PERK, IRE1, and ATF6, thereby activating these three molecules. Subsequently, the autophosphorylated PERK phosphorylates eIF2a to inhibit mRNA translation and global protein synthesize, and increases ATF4 expression. The activated IRE1 cleaves *XBP1* mRNA and the isolated ATF6 is cleaved by 1-site protease (SP1) and 2-site protease (SP2) proteins in Golgi complex. At last, the spliced Xbp1, the ATF4 and the spliced ATF6 promote the expression of ER chaperone genes, which are further involved in eliminating unfolded proteins and restoring homeostasis in normal cells (Figure 1; Ji et al., 2019). Recent studies have shown that ERS plays an important role in many diseases including metabolic diseases and inflammatory diseases, which has become a research hotspot (Wang and Kaufman, 2016).

## Overview of H_2_S

**FIGURE 2:**
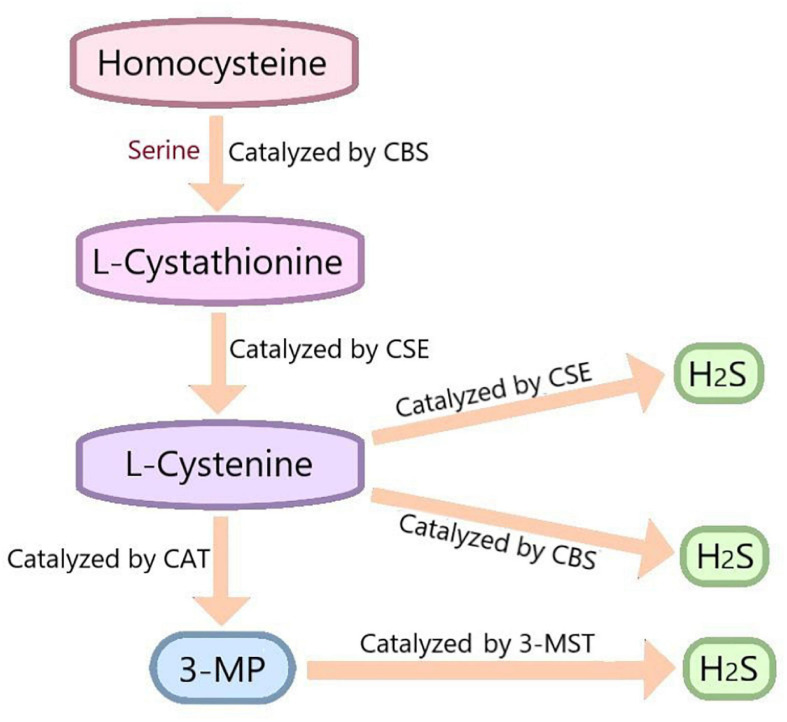
*In vivo* synthesis process of H_2_S. Cysteine is produced by the β-substitution reaction of homocysteine with serine catalyzed by CBS. CSE catalyzes the elimination of α, γ - cysteine from L-cystathionine to produce L-cystenine. Under the catalysis of CBS and CSE, L-cysteine generates H_2_S through β - elimination reaction. L-cystenine also produces 3-mercaptopyruvate (3-MP) by transferring its amines to α-ketoglutarate catalyzed by cysteine aminotransferase (CAT). 3-MST catalyzes the sulfur of 3-MP to convert into H_2_S. CBS, cystathionine-beta-synthase; CSE, cystathionine-gamma-lyase; 3-MST, 3-mercaptopyruvate thiotransferase; 3-MP, 3-mercaptopyruvate; CAT, cysteine aminotransferase.

Hydrogen sulfide has been recognized as a toxic and odorous gas for a long time. However, since the 1990s, many studies have demonstrated that together with NO and CO, H_2_S belongs to the category of gasotransmitter (Katakami, 2018). H_2_S is endogenously synthesized by three enzymes: cystathionine-β-synthase(CBS), cystathionine-γ-lyase(CSE) and 3-mercaptopyruvate thiotransferase (3-MST) (Rose et al., 2017). The distribution of these enzymes was specific. CBS is mainly expressed in the pancreas, brain and reproductive organs (Miyamoto et al., 2015; Wang et al., 2018). CSE is highly expressed in the liver, kidney, neurons, macrophages, and smooth muscle cells (Badiei et al., 2016). 3-MST is mainly expressed in red blood cells and heart (Shen et al., 2019). In the process of endogenous H_2_S production, CBS catalyzes the β-substitution reaction of homocysteine with serine to produce L-Cystathionine. L-Cystenine is produced by the elimination of α, γ-cysteine of L-Cystathionine catalyzed by CSE. L-cystenine then produces H_2_S via β elimination reaction catalyzed by CSE/CBS. L-cystenine also produces 3-mercaptopyruvate (3-MP) by transferring its amines to α-ketoglutarate catalyzed by cysteine aminotransferase (CAT). 3-MST catalyzes the sulfur of 3-MP to convert into H_2_S (Figure 2). In addition, there are some other recognized or assumed sources of H_2_S, including D-amino acid oxylase (Han et al., 2020) and methionine oxidase (Pol et al., 2018). In biological system, several non-enzymatic methods can also produce H_2_S (Yang et al., 2019).

Hydrogen sulfide can pass through the cell membrane freely, so it does not function through the receptor (Yang et al., 2005). The biological function of H_2_S mainly depends on the formation of new molecules, such as S-nitrosothiol, and its possible mechanisms include reversible protein sulfidation (Dongo et al., 2018). H_2_S plays important roles in many physiological processes, including vasodilation, blood pressure reduction (Greaney et al., 2017; Jin et al., 2017), anti-apoptosis (Li et al., 2019), anti-inflammation (Zhao et al., 2019), anti oxidative stress (Tocmo and Parkin, 2019), cell survival/death, cell differentiation, cell proliferation/hypertrophy, mitochondrial bioenergetics/biogenesis and ERS (Zhang et al., 2017). Recent studies indicate that H_2_S plays an important protective role through influencing ERS in many diseases including diabetic cardiomyopathy, neurological diseases, respiratory diseases and vascular diseases (Wang et al., 2020). However, the mechanism of H_2_S regulating ERS in disease, especially its signal transduction pathway has not been fully studied.

## The Signaling Pathways Involved in the Effects of H_2_S on Ers

More and more evidences have showed that the effects of H_2_S on ERS involve a variety of signaling pathways, including protein kinase B (Akt)-heat shock protein 90 (Hsp90) signaling pathway, brain derived neurotrophic factor(BDNF)/tyrosine kinase receptor type 2(TrkB) signaling pathway, phosphatidylinositol 3 kinase(PI3K)/protein kinase B (Akt) signaling pathway and nuclear factor kappa-B(NF-κB)/mitogen-activated protein kinase(MAPK) signaling pathway.

### Akt-Hsp90 Signaling Pathway

Heat shock protein 90 is a highly conserved ATP dependent chaperone in eukaryotic cells. It participates in protein homeostasis, plays an important role in the remodeling of many client proteins and is involved in signal transduction, protein transport and receptor maturation (Schopf et al., 2017; Radli and Rudiger, 2018). Akt, also known as PKB (protein kinase B), is an important Hsp90-dependent serine-threonine kinase, which regulates many processes of mammalian cells and plays a key role in glucose uptake, cell survival and proliferation. Dysregulation of Akt signaling is associated with many diseases, such as type 2 diabetes, cardiovascular disease and cancer (Sato et al., 2000; Gray and Coster, 2020). Xie et al. (2012) found that exogenous H_2_S could protect SH-SY5Y cells (human neuroblastoma cell) against apoptosis by inhibiting ERS induced by 6-hydroxydopamine (6-OHDA). Mechanism studies revealed that Akt inhibitor, not MEK inhibitor, notably reversed the inhibitory effect of NaHS (H_2_S donor) on ERS induced by 6-OHDA by decreasing *GRP78* mRNA expression level and phosphorylation level of eIF2α, indicating that the protective effects of exogenous H_2_S against ERS induced by 6-OHDA was achieved by stimulating Akt pathway. The suppression of Hsp90 by geldanamycin (Hsp90 inhibitor) significantly increased *GRP78* mRNA expression level. NaHS alone significantly increased Hsp90 protein expression. Pretreatment with NaHS could not decrease the upregulated *GRP78* mRNA expression level induced by geldanamycin. These suggested that Hsp90 is involved in the protective effects of H_2_S against ERS. In addition, In SH-SY5Y cells, the overexpressed CBS increased Hsp90 protein expression level and decreased eIF2α phosphorylation level, further confirming the neuroprotective effects of H_2_S against ERS are achieved through upregulation of Hsp90. Inhibition of Akt with inhibitor decreased Hsp90 protein expression level induced by exogenous H_2_S, suggesting that the activation of Akt is vital for exogenous H_2_S promotion of Hsp90 protein expression. Moreover, the interaction between Akt and Hsp90 can inhibit apoptosis to promote cell survival. From the above, it can be inferred that exogenous H_2_S exerts neuroprotective effects by inhibiting 6-OHDA-induced ERS through activating Akt-Hsp90 pathway, which needs to be further verified, such as demonstrating that the observed stress marker are dependent on the UPR transducers (Xie et al., 2012). The activation of Hsp90 by Akt induced by exogenous H_2_S remains unclear. It has been reported that H_2_S promotes insulin-secreting cells apoptosis through increasing ERS (Yang et al., 2007), which is in contradiction with the above. Whether H_2_S inhibits ERS or promotes ERS may depend on the type of tissue cells, the type and stage of pathological process.

### BDNF/TrkB Signaling Pathway

Brain derived neurotrophic factor (BDNF) is a neurotrophic factor and mainly expressed in the hippocampus and cortex to regulate the central nervous system (Binder and Scharfman, 2004; Mariga et al., 2017). It plays an important role in the development, maintenance and plasticity of the central and peripheral nervous systems (Tapia-Arancibia et al., 2008; Bath and Lee, 2010; Lu et al., 2014). BDNF has a significant neuroprotective effect by binding to its specific receptor TrkB (Massa et al., 2010; Pandya et al., 2013). It has been reported that the inhibition of ERS contributes to the neuroprotective effects mediated by BDNF (Shimoke et al., 2004; Chen et al., 2007). Wei et al. (2014) found that the expression level of BDNF in hippocampus of rats treated with Homocysteine (Hcy) decreased significantly, while exogenous H_2_S could reverse the change. Hcy notably promoted the expression level of GRP78, CHOP and cleaved caspase-12 in the hippocampus of rats, while pretreatment with NaHS before Hcy treatment for 7 days significantly reversed these changes. These findings suggested exogenous H_2_S could inhibit Hcy-induced ERS and neuronal apoptosis. The similar results were obtained *in vitro* with PC12 cells. The inhibition of BDNF/TrkB pathway with TrkB inhibitor (k252a) reversed the inhibitory effects of exogenous H_2_S on Hcy-induced ERS and apoptosis in rat hippocampus, indicating that exogenous H_2_S played a neuroprotective role against Hcy-induced ERS through promoting BDNF/TrkB pathway (Wei et al., 2014). Studies have showed that depress can suppress the expression level of BDNF in the hippocampus, whereas antidepressants has the opposite effect (Castren and Kojima, 2017), suggesting that BDNF is involved in the development of depress. Recent evidences showed that exogenous H_2_S activated the BDNF/TrkB pathway by increasing the expression levels of BDNF and p-TrkB in the hippocampus of chronic unpredictable mild stress (CUMS)-induced rats. CUMS could increase the expression levels of GRP78, CHOP, and cleavage caspase-12 in the hippocampus of rats, while NaHS could reverse the changes, indicating that exogenous H_2_S inhibited CUMS-induced ERS. The suppression of BDNF-TrkB pathway with TrkB inhibitor (k252a) reversed the antidepressant-like efects of exogenous H_2_S against hippocampal CUMS-induced ERS in the CUMS-exposed rats, indicating that BDNF-TrkB pathway mediated the antidepressant effects of exogenous H_2_S (Wei et al., 2018). BDNF/TrkB pathway may be an important target of H_2_S related drugs in the treatment of nerve injury. The mechanism of H_2_S improving nerve injury through BDNF/TrkB pathway and ERS-inducing nerve injury need further investigation, which may contribute to the development of anti-nerve injury drugs of H_2_S.

### PI3K/Akt Signaling Pathway

PI3K/Akt signaling pathway is important for cell growth and survival under physiological and pathological conditions (Porta et al., 2014; de Melo et al., 2017) and plays an important role in mediating mesenchymal stem cells (MSCs) survival signal transduction (Mangi et al., 2003). The activation of this pathway leads to dysfunction of cell function control, eventually leading to competitive growth advantage, angiogenesis and therapeutic resistance. New drugs targeting PI3K/Akt signaling pathway can improve the current therapeutic effects through improving selectivity and potency, as well as combination with other treatment strategies (Wu et al., 2018a). Guo et al. (2015) found that endogenous H_2_S could improve the survival rate of transplanted mesenchymal stem cells (MSCs), in which PI3K/Akt signaling pathway was involved. Hypoxia and serum deprivation (H/SD) could significantly induce MSCs apoptosis, leading to poor graft survival rate. The overexpression of CSE/H_2_S system could prevent H/SD-induced reduction of endogenous H_2_S production and protect MSCs against apoptosis. Mechanism studies revealed that the CSE overexpression counteracted the increased expression levels of CHOP and GRP78 induced by H/SD and promoted the phosphorylation level of Akt in MSCs, indicating that endogenous H_2_S suppressed ERS and activated PI3K/Akt signaling pathway, and ERS and PI3K/Akt signaling pathway might be involved in endogenous H_2_S protection of MSCs against H/SD-induced apoptosis (Guo et al., 2015). ERS can induce apoptosis (Hu et al., 2019) and Genipin can inhibit ERS through activating the PI3K/Akt signaling pathway (Luo et al., 2019). Therefore, it can be inferred that endogenous H_2_S suppresses ERS to protect MSCs against H/SD-induced apoptosis by activating PI3K/Akt signaling pathway, which needs to be further investigated. The PI3K/Akt signaling pathway is also involved in the improvement of intervertebral disc degeneration (IVDD) by H_2_S. It has been reported that ERS-induced apoptosis is related with disc cell apoptosis of IVDD in rats (Zhao et al., 2010). IL-1β could induce apoptosis and decrease H_2_S generation in nucleus pulposus (NP) cells, while the inhibition of CBS or CSE with siRNA notably increased IL-1β-induced apoptosis of NP cells. Moreover, exogenous H_2_S could decrease the level of IL-1β and apoptosis in NP cells. Therefore, it can be inferred that H_2_S can inhibit IL-1β-induced apoptosis of nucleus pulposus (NP) cells. Exogenous H_2_S could inhibit ERS induced by IL-1β, indicating that exogenous H_2_S could suppress apoptosis of NP cells to improve IVDD through ERS. Mechanism studies revealed that in NP cells, exogenous H_2_S could activate the PI3K/Akt signaling pathway, while inhibiting the PI3K/Akt signaling pathway with inhibitors (LY294002) could partially abolish the above protective effects of H_2_S on apoptosis of NP cells, indicating that exogenous H_2_S improved IVDD through suppressing ERS by activating PI3K/Akt signaling pathway (Xu et al., 2017), which needs to be further studied. For example, the relationship between ERS and IL-1β needs to be further researched. Contrary to the above, H_2_S can promote cisplatin-induced nephrotoxicity of mice, possibly by inducing apoptosis (Liu et al., 2016). This contradiction may be due to the different stimulation concentration and duration of H_2_S. Whether H_2_S can promote ERS-induced apoptosis through inhibiting PI3K/Akt signaling pathway remains to be studied. PI3K/Akt signaling pathway will provide a new target for H_2_S to improve IVDD.

### NF-κB/MAPK Signaling Pathway

Mechanical ventilation is a rescue intervention for acute respiratory failure, which can cause local and systemic inflammatory response, and lead to ventilator-induced lung injury (VILI). VILI is an important cause of death in patients with respiratory failure (Wienhold et al., 2018). Exogenous H_2_S has been reported to improving VILI by mitigating the histopathological damage, pulmonary edema, lung permeability, inflammation, apoptosis and oxidative damage of lung tissue. In the model of VILI, the protein expression levels of GRP94, GRP78, p-PERK, p-eIF2α, p-IRE1α, nuclear ATF4, the apoptosis related proteins and the pro-inflammatory factors were increased significantly, and were decreased notably by NaHS treatment, indicating that exogenous H_2_S could suppress VILI induced-ERS, apoptosis to improve VILI (Ge et al., 2019). ERS can induce apoptosis and inflammation (Xiao et al., 2016; Zheng et al., 2018), so, it can be inferred that exogenous H_2_S alleviates VILI through improving apoptosis and inflammation by inhibiting ERS. NF-κB is an inducible transcription factor, which plays an important role in immune response, inflammatory response, cell differentiation and survival of normal and malignant cells (Wienhold et al., 2018). MAPK plays a key role in transforming extracellular stimulation into a variety of cellular responses which includes cell growth, migration, proliferation, differentiation and apoptosis (Ge et al., 2019). Studies showed that in the model of VILI, the phosphorylation levels of nuclear NF-κB, p65, MAPK p38, JNK, and ERK were all increased, which were decreased significantly by ERS inhibitor(4-PBA) or NaHS, suggesting that VILI activates the NF-κB/MAPK signaling pathway, which is reversed by exogenous H_2_S or 4-PBA (Ge et al., 2019). Therefore, it can be inferred that exogenous H_2_S may alleviate VILI by inhibiting ERS, in which NF-κB/MAPK signaling pathway is involved. The above inference needs to be further confirmed by the use of related inhibitors, such as the inhibitor of NF-κB/MAPK signaling pathway.

## Conclusion

**FIGURE 3:**
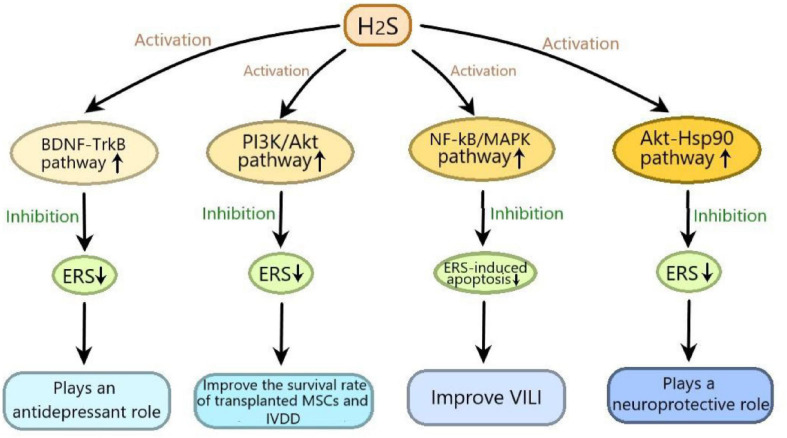
The signaling pathways involved in the effects of hydrogen sulfide on endoplasmic reticulum stress. H_2_S exerts neuroprotective effect by inhibiting 6-hydroxydopamine induced ERS through activating Akt-Hsp90 signaling pathway; H_2_S plays an antidepressant role through inhibiting ERS by activating BDNF-TrkB signaling pathway; H_2_S improves the survival rate of transplanted mesenchymal stem cells and IVDD by suppressing ERS-induced apoptosis through activating PI3K/Akt signaling pathway; H_2_S alleviates VILI by inhibiting ERS through suppressing NF-κB/MAPK signaling pathway. ERS, endoplasmic reticulum stress; Akt-Hsp90, protein kinase B-heat shock protein 90; BDNF-TrkB, brain derived neurotrophic factor-tyrosine kinase receptor type 2; PI3K/Akt, phosphatidylinositol 3 kinase/protein kinase B; NF-κB/MAPK, nuclear factor kappa-B/mitogen-activated protein kinases; VILI, ventilator-induced lung injury; IVDD, intervertebral disc degeneration.

It has been reported that H_2_S plays an important role in many diseases by regulating ERS, in which many signaling pathways are involved. In this review, we summarized as follows: (1) H_2_S exerts neuroprotective effect by inhibiting 6-OHDA-induced ERS through activating Akt-Hsp90 signaling pathway; (2) H_2_S plays an antidepressant role through inhibiting ERS by activating BDNF-TrkB signaling pathway; (3) H_2_S improves the survival rate of transplanted MSCs and IVDD by suppressing ERS-induced apoptosis through activating PI3K/Akt signaling pathway; (4) H_2_S alleviates VILI by inhibiting ERS through suppressing NF-κB/MAPK signaling pathway (Figure 3). These signaling pathways may provide promising targets for the treatment of many diseases, including nervous system diseases, depression, IVDD and VILI. Moveover, H_2_S-regulated ERS plays an important protective role in diabetic cardiomyopathy, respiratory diseases and vascular disease (Wang et al., 2020), so more studies are needed to determine whether these signaling pathways are involved in H_2_S-regulated ERS in these diseases. Furthermore, in addition to the abovementioned signaling pathways, new signaling pathways need to be elucidated in H_2_S-regulated ERS in various disease models.

The existing H_2_S releasers cannot fully meet the needs of the research and development of H_2_S related drugs, so the development of new H_2_S releasers is of great significance for the application of H_2_S related drugs in clinical disease treatment.

In conclusion, with the in-depth study of the signal pathways involved in the effects of H_2_S on ERS in diseases and the discovery of new H_2_S releasing agents, it will provide a new strategies for research and disease treatment of H_2_S-related medicine.

## Author Contributions

HW devised, wrote, and financially supported the manuscript. SZ wrote and financially supported the manuscript. XnL and MS contributed to the writing. XaL contributed to the drawing. All authors contributed to the article and approved the submitted version.

## Conflict of Interest

The authors declare that the research was conducted in the absence of any commercial or financial relationships that could be construed as a potential conflict of interest.
